# Disentangling genetic from environmental effects on phenotypic variability of southern rock lobster (*Jasus edwardsii*) postlarvae

**DOI:** 10.1002/ece3.9519

**Published:** 2022-11-16

**Authors:** Cecilia Villacorta‐Rath, Bridget S. Green, Caleb Gardner, Nick P. Murphy, Carla A. Souza, Jan M. Strugnell

**Affiliations:** ^1^ Centre for Tropical Water and Aquatic Ecosystem Research (TropWATER) James Cook University Townsville Queensland Australia; ^2^ Institute for Marine and Antarctic Studies University of Tasmania Hobart Tasmania Australia; ^3^ Department of Ecology, Environment and Evolution La Trobe University Bundoora Victoria Australia; ^4^ Centre for Sustainable Tropical Fisheries and Aquaculture James Cook University Townsville Queensland Australia

**Keywords:** dispersal history, phenotypic variability, pueruli, recruitment, size‐at‐settlement

## Abstract

Environmental conditions experienced during the larval dispersal of marine organisms can determine the size‐at‐settlement of recruits. It is, therefore, not uncommon that larvae undergoing different dispersal histories would exhibit phenotypic variability at recruitment. Here, we investigated morphological differences in recently settled southern rock lobster (*Jasus edwardsii*) recruits, known as pueruli, along a latitudinal and temporal gradient on the east coast of Tasmania, Australia. We further explored whether natural selection could be driving morphological variation. We used double digest restriction site‐associated DNA sequencing (ddRADseq) to assess differences in the genetic structure of recently settled recruits on the east coast of Tasmania over 3 months of peak settlement during 2012 (August–October). Phenotypic differences in pueruli between sites and months of settlement were observed, with significantly smaller individuals found at the northernmost site. Also, there was a lack of overall genetic divergence; however, significant differences in pairwise F_ST_ values between settlement months were observed at the southernmost study site, located at an area of confluence of ocean currents. Specifically, individuals settling into the southernmost earlier in the season were genetically different from those settling later. The lack of overall genetic divergence in the presence of phenotypic variation indicates that larval environmental history during the dispersal of *J. edwardsii* could be a possible driver of the resulting phenotype of settlers.

## Graphical abstract

We found that *Jasus edwardsii* settlers exhibit phenotypic variability across a geographical and temporal gradient; however, this is not correlated with genetic divergence. Given the protracted pelagic larval duration of the species, we hypothesize that dispersal history drives phenotypic variability in this species.

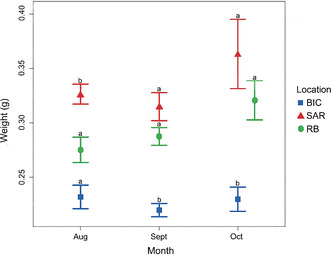

## 1 INTRODUCTION

Genetic connectivity in many marine invertebrates is contingent on the dispersal of pelagic larvae (Hedgecock, 1986). Highly structured populations typically result from larvae with short pelagic durations or nonplanktonic larval stages, whereas species with long pelagic larval durations (PLDs) have shown little genetic structure across large geographical scales (Sandoval‐Castillo et al., 2018; Villacorta‐Rath et al., 2016; Watts & Thorpe, 2006). Long PLDs, however, could lead to reduced recruitment success due to the larger window of time where predation and starvation can act upon individuals (Morgan, 2020). Although it has been proven that fast growth promotes larval survival (Crean et al., 2011; Marshall et al., 2010), the driver of phenotypic variability is a complex interaction of maternal fecundity, maternal energy investment in the offspring and environmental factors (Cobb et al., 1997; González‐Ortegón & Giménez, 2014; Green et al., 2014).

Indeed, the environment has profound effects on the metabolism and growth of larvae, which can be carried over throughout their life history (Marshall et al., 2003). Cohorts of marine invertebrate recruits exhibit high phenotypic divergence due to the wide range of environmental conditions experienced during the pelagic larval stage (PLD) (Rey et al., 2016). For example, if egg‐hatching coincides with low food availability, larvae may experience poor survival or high postsettlement mortality (Giménez, 2006). Also, the offspring of females that become reproductive at different times of the reproductive season will have slightly different phenotypes at settlement, probably because of the environmental history experienced during dispersal (Giménez, 2006; Kunisch & Anger, 1984). Although evidence suggests the existence of phenotypic differences in invertebrate recruits across geographic and temporal scales (reviewed by Sanford & Kelly, 2011), it is unknown whether these differences are purely driven by dispersal history or if local adaptation also plays an important role in shaping recruitment (Hedgecock, 1986).

The present study explored the link between phenotype and genotype using the southern rock lobster, *Jasus edwardsii*, as a model species given its large reproductive output and protracted pelagic larval duration. This species is highly fecund, producing approximately 500,000 eggs per brood (Green et al., 2014). Adults experience seasonal reproduction that extends from May to September, but egg‐hatching periods vary slightly across populations through a latitudinal gradient (Powell, 2016). *Jasus edwardsii* exhibits the largest PLD of all species within the Palinuridae family, spending between 12 and 24 months before metamorphosing into a puerulus postlarva (Booth, 1994), giving a large window for environmental factors to shape larval phenotypes. Recruitment monitoring of this species in southeast Australia has evidenced large variability in abundance (Hinojosa et al., 2017; Linnane et al., 2014) and in size‐at‐settlement (Booth, 1979, 1994), as it is expected for highly fecund species (Cobb et al., 1997). Additionally, sweepstakes reproductive success and natural selection have been proposed as possible mechanisms driving chaotic genetic patchiness in this species (Villacorta‐Rath et al., 2018).

Based on previous evidence of phenotypic and genetic differences in recruits, we hypothesized that dispersal history drives phenotypic variability of recently settled individuals. The present study explored patterns of morphological and genetic differences in recently settled *J. edwardsii* along a latitudinal and temporal gradient in Tasmania, Australia. More specifically, the aims of this study were: (1) to compare size‐at‐settlement of *J. edwardsii* pueruli at three sampling sites during three consecutive settlement months, (2) to determine genetic differences in recently settled *J. edwardsii* across a geographical and temporal gradient, and (3) to investigate if the phenotypic variation was related to neutral and non‐neutral genotypic variation.

## 2 MATERIALS AND METHODS

### 2.1 Sample collection

**FIGURE 1:**
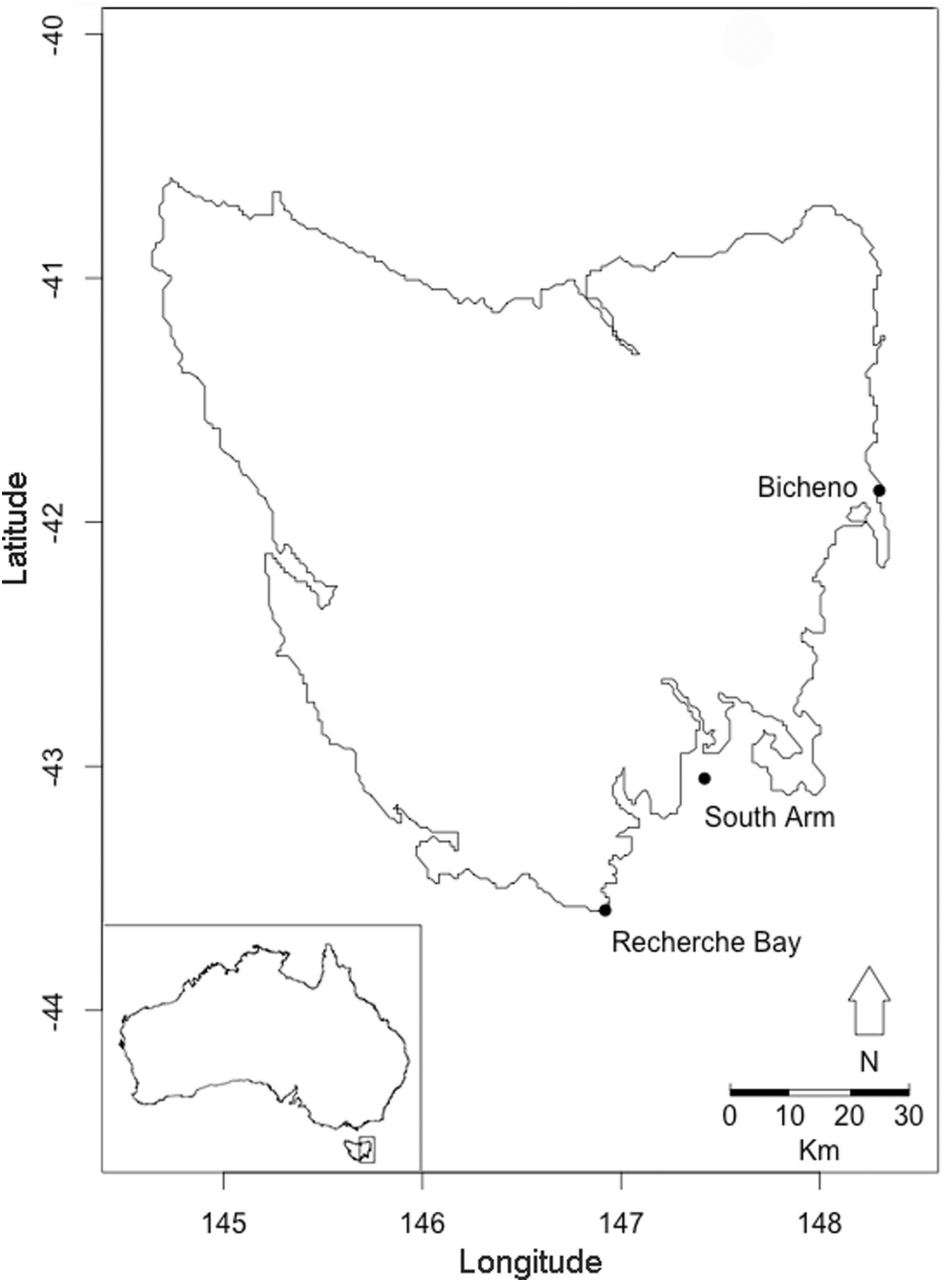
Puerulus collector sites in Tasmania, Australia

Lobster pueruli were caught in crevice collectors (Booth & Tarring, 1986) deployed at three sites on the east coast of Tasmania, southeast Australia (Figure 1) (Gardner et al., 2000). Collectors were attached to permanent structures fixed on sandy substratum at three to nine meters of depth (Gardner et al., 2000). Puerulus collectors were serviced monthly by a diver who placed a mesh bag around each collector and attached a rope with a buoy to each bag. Collectors were then hauled into the boat, where they were cleaned (Gardner et al., 2000). All pueruli were removed from the collectors and immediately stored in 90% ethanol. For the purpose of this study, only recently settled pueruli (stages 1 and 2) during the months of August, September, and October 2012 were considered (Table 1).

**TABLE 1:** Number of individuals analyzed per month of collection during the year 2012 at each collector site. Number of individuals used for morphometric analyses (N_morph_) and number of individuals used for sequencing (N_seq_) are reported.

Collector site	Latitude	Longitude	Month of collection	*N*_morph_	*N*_seq_
Bicheno	41°52′ S	148°18′ E	August	19	14
			September	17	7
			October	15	15
South Arm	43°03′ S	147°25′ E	August	20	16
			September	12	10
			October	3	4
Recherche Bay	43°35′ S	146°55′ E	August	15	11
			September	13	12
			October	13	15

*Note*: During October, morphometrics from one individual in the South Arm and two individuals in Recherche Bay could not be measured due to missing body parts.

### 2.2 Comparing pueruli size‐at‐settlement

Pueruli individuals were removed from ethanol, blot dried to eliminate the excess ethanol, and weighed (g ± 0.01). To determine whether pueruli weight varied with settlement month, site, and the interaction of both, a two‐way analysis of variance (ANOVA) was performed using the function “aov” implemented within the R package stats v4.2.1 (R Core Team, 2022). A post hoc examination of differences between individual weights was carried out using the Tukey's “honest significance difference” method using the function “TukeyHSD” of the R package stats v4.2.1.

### 2.3 DNA extraction and ddRADseq library preparation

DNA was extracted from tissue from the horns and legs of each individual using the DNeasy Blood and Tissue kit (Qiagen) following the manufacturer's instructions. DNA concentration was quantified on a Qubit® 2.0 Fluorometer (Life Technologies). DNA integrity was determined through gel electrophoresis to verify that high molecular weight DNA was obtained in all samples (>1000 base pairs [bp]).

Four ddRADseq libraries were prepared using a modified version of Peterson et al. (2012) ddRAD protocol, described in Villacorta‐Rath et al. (2016). Gel size selection was set to 400–600 bp in order to maximize the overlapping region among all ddRAD libraries and therefore increase the chances of encountering common loci. A total of 59–60 samples were pooled in each library. Additionally, within each library, 9–10 technical replicates were included in order to test for sequencing‐derived differences between libraries (Mastretta‐Yanes et al., 2015). Electrophoretic assays of pooled libraries were run using an Agilent BioAnalyzer (Agilent Technologies) to determine their exact molecular weight range and library concentration. Pooled libraries were sequenced at the Australian Genome Research Facility (AGRF) on the Illumina HiSeq 2500 platform using a 100 bp single‐end kit. After demultiplexing, 28 samples that yielded a lower‐than‐average number of reads were resequenced in half a lane of the Illumina HiSeq 2500 platform using a 100 bp single‐end kit.

### 2.4 Analyses of raw sequencing data and reference catalog building

An initial quality check of raw indexed data was performed using FastQC v0.10.1. Data were then demultiplexed using the *“*process_radtags” protocol from Stacks v1.29 (Catchen et al., 2011) and hard trimmed to 75 bp to ensure that Phred Quality Score (Q Score) of all reads was above 30. Finally, demultiplexed libraries were filtered for bacterial and viral content using Kraken‐gcc v0.10.4 (Wood & Salzberg, 2014).

Filtered reads were processed through a custom‐built “rad‐loci” pipeline (https://github.com/molecularbiodiversity/rad‐loci) to obtain a catalog of reference loci detailed within Villacorta‐Rath et al. (2016). An initial run of the rad‐loci pipeline, read alignment, and SNP calling was performed and samples with more than 25% missing data were discarded in order to maximize the final number of polymorphic loci obtained. A total of 70 samples were discarded and 193 samples with less than 25% missing data remained. The subsequent rad‐loci pipeline run was therefore performed with a subset of 193 samples using VSearch v1.1.3 (https://github.com/torognes/vsearch). Initially, all reads were pooled and clusters with a depth of at least 193 were retained, assuming that there would be at least one read per sample represented in each cluster. Subsequently, all clusters that were comprised of reads with more than a 4% mismatch (3 bp) were discarded. Assuming that each member of a cluster was an allele, only clusters that had between two and 16 members were retained. Finally, only samples with a minimum of 10,000 alleles and 30% missing data were retained for subsequent read alignment and SNP calling.

### 2.5 Read alignment, variant calling, and SNP filtering

Individual filtered reads of all samples were aligned to the reference catalog using bwa‐intel v0.7.12 (Li, 2013). Variant calling was performed using the Genome Analysis Toolkit (GATK) v3.3_0 (McKenna et al., 2010), with a further correction of the variant call format (vcf) file performed to ensure the accuracy of the reference and alternate allele calls, and to filter out false positives. In the absence of a reference genome, the correction was made by calculating the ratio between the highest quality score over depth (“QD” in vcf file) and the lowest QD. If one sample at a specific position had a ratio threshold of 10, which corresponds to a 10% error on a Phred scale, the SNP at that position was replaced with missing data.

SNP filtering was performed in vcftools‐gcc v0.1.13 to ensure that only bi‐allelic data were present (−‐min‐alleles 2, −‐max‐alleles 2), to remove SNPs that were potentially in linkage disequilibrium (−‐min‐r2 0.2), to discard SNPs with a minor allele frequency (MAF) of less than 5% (−‐maf 0.05), and to ensure that the minimum SNP depth was 5 (−‐minDP 5). The maximum amount of missing data for each locus was set to 25% (−‐max‐missing 0.75) and individuals with more than 25% missing data were removed from subsequent analyses. Finally, only one SNP per locus (−‐thin 75) was retained.

A total of 22 technical replicates remained after the removal of individuals with more than the missing data threshold. A Principal Component Analysis (PCA) was performed to visualize the spatial distribution of replicated samples using the function "dudi.pca" of the R package ade4 v1.7‐19 (Dray & Dufour, 2007).

### 2.6 Outlier SNP identification

Two outlier identification methods were used in order to minimize false positives in the panel of SNPs under putative positive selection. The first SNP characterization was performed in the software Lositan (Antao et al., 2008) using 100,000 simulations, a confidence interval of 0.95, and a false discovery rate of 0.1 (Jacobsen et al., 2014). The second SNP characterization was carried out using the R package OutFLANK v0.2 (Whitlock & Lotterhos, 2015). The proportion of loci trimmed from both tails of the F_ST_ distribution was set to 5% (LeftTrimFraction = 0.05, RightTrimFraction = 0.05), the minimum heterozygosity required before including calculations from a locus was set to 0.1 (Hmin = 0.1), and the false discovery rate was 0.1 (qthreshold = 0.1). Only SNPs characterized as being under putative positive selection by both software packages were retained for downstream analyses.

Analysis of isolation by time requires the use of neutral markers that are under Hardy–Weinberg equilibrium (HWE) (Hendry & Day, 2005). Therefore further filtering of the neutral SNP panel was performed in vcftools‐gcc v0.1.13 using the function ‐‐hwe 0.01.

To determine whether the loci containing SNPs putatively under positive selection were contained in protein‐coding regions, sequences were BLASTed against the complete *Homarus americanus* transcriptome (McGrath et al., 2016) and a *J. edwardsii* transcriptome database (SRA Bioproject accession number: PRJNA386609) using BLAST+ v2.2.29. Queries with statistically significant e‐values (E < 10^−6^) and more than 84% identity were considered as valid alignments. Transcriptome sequences that provided significant alignments were annotated using the Trinotate pipeline (https://trinotate.github.io/) to determine whether they aligned with any known protein domain.

### 2.7 Analyses of genetic diversity

Global F_ST_ values and confidence intervals for each SNP panel were estimated using the R package mmod v1.3.2 (Winter, 2012). Additionally, pairwise F_ST_ values between sampling sites and years as well as confidence intervals were calculated in the R package hierfstat v0.04‐22 (Goudet, 2005). A false discovery rate correction (FDR) was applied to calculated *p*‐values using the function “p.adjust” of the R package stats v4.2.1 (R Core Team, 2022). Additionally, a discriminant analysis of principal components (DAPC) and a PCA were performed to determine the possible number of genetic clusters based on allele frequencies of all three sampling sites across three settlement months using the R packages adegenet v2.1.8 (Jombart, 2008) and ade4 v1.7‐19 (Dray & Dufour, 2007).

Analyses of Molecular Variance (AMOVA) (Excoffier et al., 1992) were performed in order to assess the amount of genetic variance explained by (1) settlement month, and sampling site using the R packages poppr v2.9.3 (Kamvar et al., 2013) and ade4 v1.7‐19 (Dray & Dufour, 2007).

Genetic diversity was quantified using the standardized individual heterozygosity (sh). This metric represents the proportion of heterozygous loci over the mean heterozygosity across all markers, so that heterozygosity of all individuals is measured on the same scale (Coltman et al., 1999). The standardized individual heterozygosity was calculated for both SNP panels at (1) each site with all three settlement months combined and (2) at each settlement month at each site using the R package Rhh v1.0.1 (Alho et al., 2010). Differences between the maximum and minimum values of standardized individual heterozygosity were examined using a Mann–Whitney test. A Bonferroni correction was applied in order to account for multiple comparisons, and α was set at the 0.05/4 level.

### 2.8 Isolation by time

Isolation by time (IBT) was estimated using Mantel tests between temporal and genetic distance using 9999 permutations within the R package ade4 v1.7‐19 (Dray & Dufour, 2007). The function “dist.gene” was used to compute the genetic pairwise distance between samples using the R package ape v5.6‐2 (Paradis et al., 2004). The pairwise distance between two individuals is the number of loci for which they differ, divided by the total number of loci (Paradis et al., 2004). The temporal distance matrix was calculated using the Euclidean distance of days between samples in the R package vegan v2.6‐4 (Oksanen et al., 2022).

## 3 RESULTS

### 3.1 Differences in time and size‐at‐settlement

**FIGURE 2:**
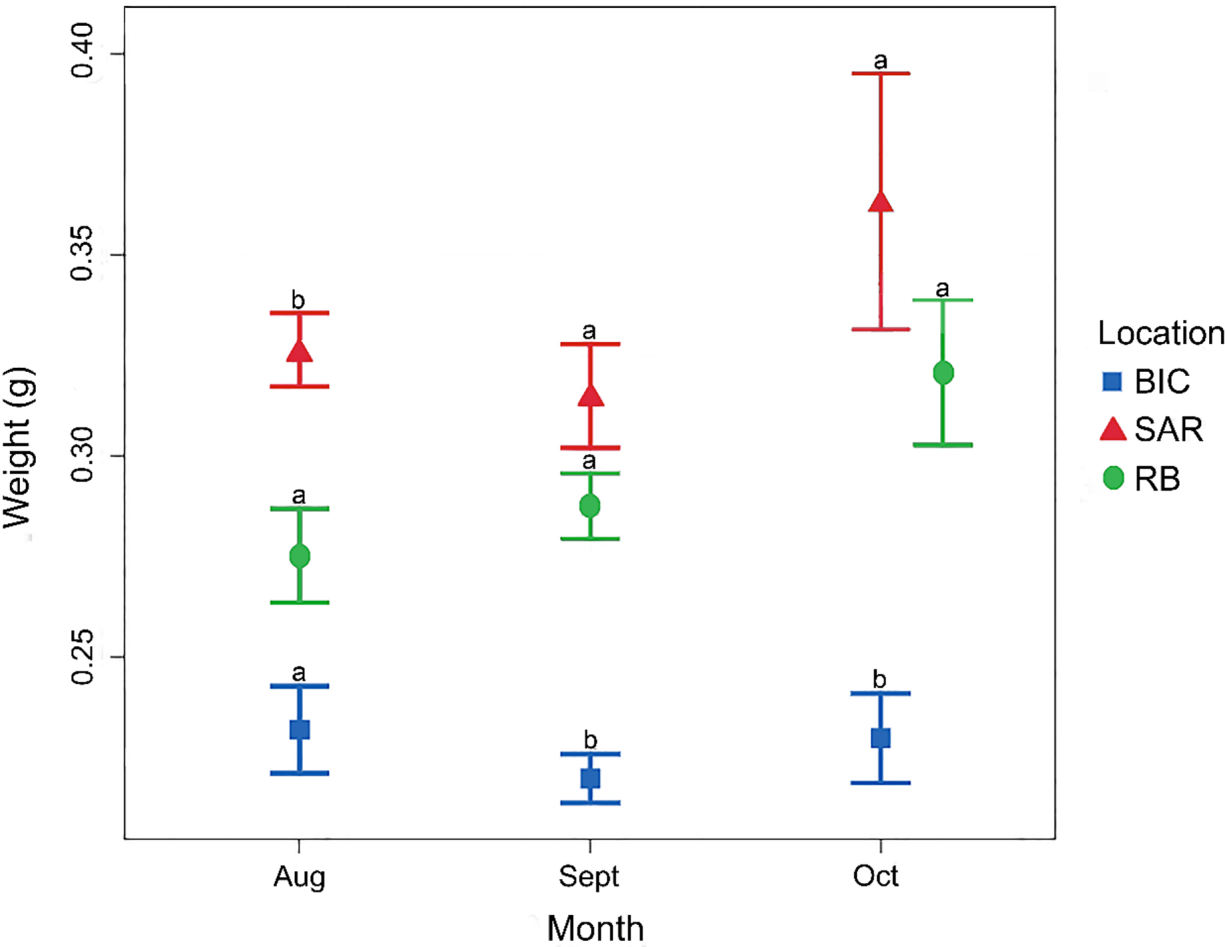
Average individual weight (±SE) of pueruli settling into Bicheno (BIC), South Arm (SAR), and Recherche Bay (RB) during 3 months of study in 2012. Significant differences between sites during each month are denoted with letters.

The weight of individual puerulus differed significantly between sites and settlement months throughout the 2012 winter settlement peak. However, the interaction between the settlement site and month did not differ significantly (Table 2). Pueruli were lighter in weight in the most northerly site, Bicheno, and heavier at the midsampling site, South Arm (Figure 2; Table 3).

**TABLE 2:** Comparison of effects of settlement site, month, and the interaction of both on pueruli weight‐at‐settlement

Variable	df	MS	*F*	*p*
Site	2	0.10975	57.753	**<2 e‐16**
Month	2	0.00593	3.122	**.0477**
Site*Month	4	0.00257	1.352	.2548
Error	118	0.00190		

*Note*: Significant differences are in bold.

**TABLE 3:** Differences between mean individual weight per month and site of settlement and confidence intervals at a 95% confidence level based on the “Tukey's Honest Significant Difference” method.

Site (settlement month)	Mean difference	Lower limit	Upper limit	*p*‐adj
RB (Aug) ‐ BIC (Aug)	0.0432	−0.0044	0.0908	0.1066
SAR (Aug) ‐ BIC (Aug)	0.0944	0.0503	0.1385	0.0000
BIC (Sept) ‐ BIC (Aug)	−0.0121	−0.0581	0.0339	0.9957
RB (Sept) ‐ BIC (Aug)	0.0556	0.0060	0.1052	0.0160
SAR (Sept) ‐ BIC (Aug)	0.0829	0.0321	0.1337	0.0000
BIC (Oct) ‐ BIC (Aug)	−0.0021	−0.0497	0.0455	1.0000
RB (Oct) ‐ BIC (Aug)	0.0887	0.0391	0.1382	0.0000
SAR (Oct) ‐ BIC (Aug)	0.1312	0.0456	0.2168	0.0001
SAR (Aug) ‐ RB (Aug)	0.0512	0.0041	0.0982	0.0223
BIC (Sept) ‐ RB (Aug)	−0.0553	−0.1041	−0.0065	**0.0141**
RB (Sept) ‐ RB (Aug)	0.0124	−0.0398	0.0646	0.9979
SAR (Sept) ‐ RB (Aug)	0.0397	−0.0137	0.0930	0.3219
BIC (Oct) ‐ RB (Aug)	−0.0453	−0.0956	0.0050	0.1127
RB (Oct) ‐ RB (Aug)	0.0454	−0.0068	0.0976	0.1419
SAR (Oct) ‐ RB (Aug)	0.0880	0.0009	0.1751	0.0458
BIC (Sept) ‐ SAR (Aug)	−0.1065	−0.1519	−0.0611	**0.0000**
RB (Sept) ‐ SAR (Aug)	−0.0388	−0.0879	0.0103	0.2433
SAR(Sept) ‐ SAR (Aug)	−0.0115	−0.0618	0.0388	0.9984
BIC (Oct) ‐ SAR (Aug)	−0.0965	−0.1436	−0.0494	**0.0000**
RB (Oct) ‐ SAR (Aug)	−0.0057	−0.0548	0.0433	1.0000
SAR (Oct)‐SAR (Aug)	0.0368	−0.0485	0.1221	0.9086
RB (Sept) ‐ BIC (Sept)	0.0677	0.0169	0.1184	**0.0016**
SAR (Sept) ‐ BIC (Sept)	0.0950	0.0431	0.1469	**0.0000**
BIC (Oct) ‐ BIC (Sept)	0.0100	−0.0388	0.0588	0.9993
RB (Oct) ‐ BIC (Sept)	0.1008	0.0500	0.1515	**0.0000**
SAR (Oct) ‐ BIC (Sept)	0.1433	0.0571	0.2296	**0.0000**
SAR (Sept) ‐ RB (Sept)	0.0273	−0.0278	0.0825	0.8216
BIC (Oct) ‐ RB (Sept)	−0.0577	−0.1099	−0.0055	**0.0188**
RB (Oct) ‐ RB (Sept)	0.0331	−0.0210	0.0871	0.5915
SAR (Oct) ‐ RB (Sept)	0.0756	−0.0126	0.1639	0.1560
**BIC (Oct) ‐ SAR (Sept)**	**−0.0850**	**−0.1384**	**−0.0316**	**0.0001**
RB (Oct) ‐ SAR (Sept)	0.0058	−0.0494	0.0609	1.0000
SAR (Oct) ‐ SAR (Sept)	0.0483	−0.0406	0.1373	0.7345
**RB (Oct) ‐ BIC (Oct)**	**0.0908**	**0.0386**	**0.1430**	**0.0000**
**SAR (Oct) ‐ BIC (Oct)**	**0.1333**	**0.0462**	**0.2205**	**0.0001**
SAR (Oct) ‐ RB (Oct)	0.0426	−0.0457	0.1308	0.8419

*Note*: Values in bold are significant after adjustment for multiple comparisons.

### 3.2 Sequencing data and SNP characterization

**FIGURE 3:**
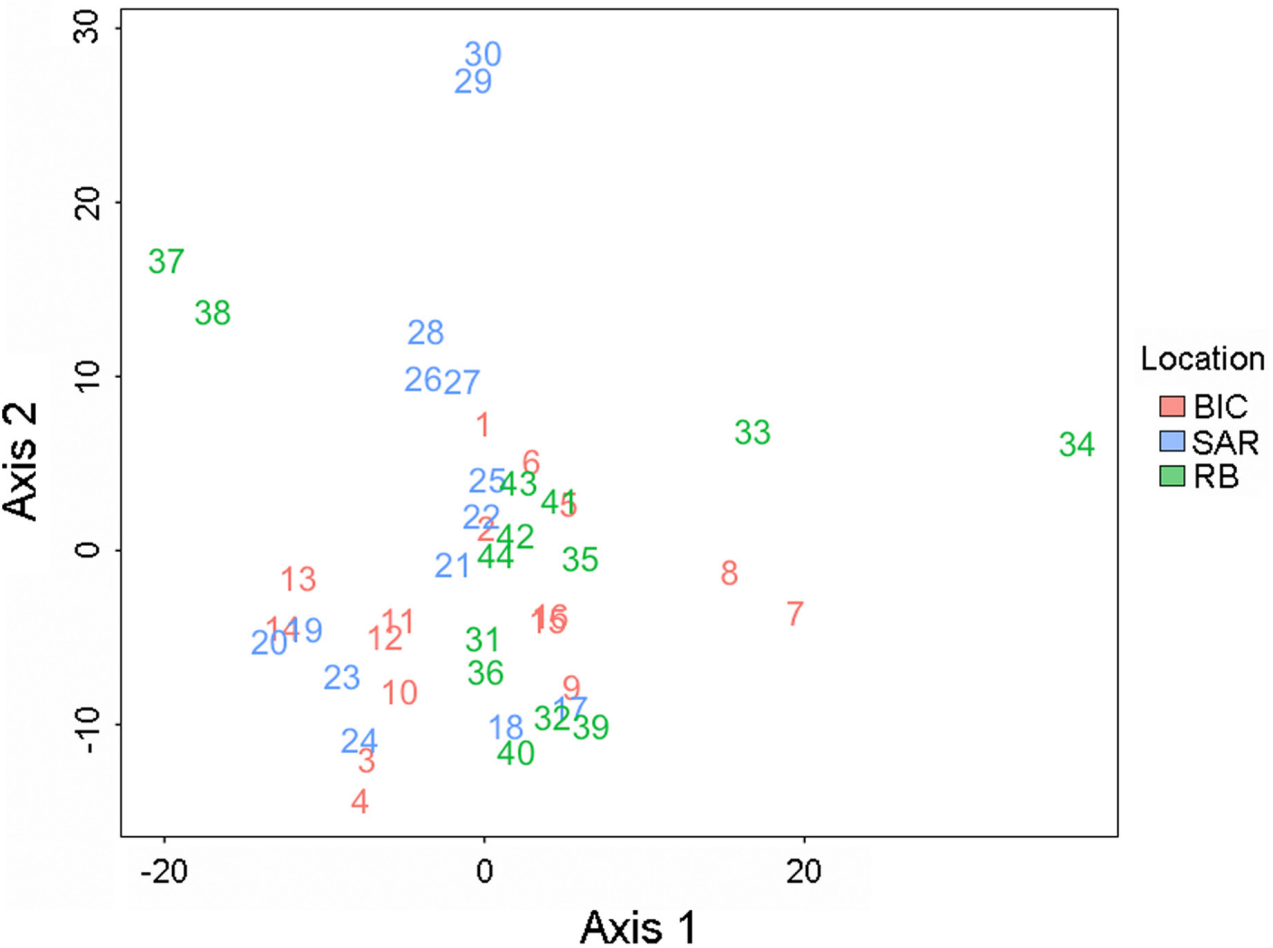
Principal component analysis plot based on allele frequencies of all 22 replicate pairs from Bicheno (red numbers), South Arm (blue numbers), and Recherche Bay (green numbers). Consecutive numbers belong to each technical replicate pair.

Sequencing of four and a half Illumina HiSeq 2500 lanes yielded a total of 757.7 million reads, with an average of 3 million reads per sample. The reference catalog yielded 9506 loci. After read alignment and variant calling, a total of 10,956 variable sites were obtained. After selecting one SNP per locus, filtering for MAF, LD, and missing data, the final number of SNPs was 1194. The PCA plot showed each intralibrary technical replicate pair to be distributed close to one another (Figure 3). This indicates that sequencing samples across multiple lanes did not introduce a large bias in the catalog‐building process.

The SNP outlier identification performed in LOSITAN produced a panel of 85 SNPs under putative positive selection, 506 SNPs under putative balancing selection, and 603 neutral SNPs. OutFLANK detected only two SNPs under putative positive selection, zero SNPs under putative balancing selection, and 1192 neutral SNPs. The final panel of 603 neutral SNPs and two SNPs under putative positive selection comprised loci identified by both software packages. HWE filtering of the neutral SNP panel detected an additional 190 SNPs not under HWE. Therefore, the final panel of neutral markers was composed of 413 SNPs.

### 3.3 Analyses of genetic diversity

**FIGURE 4:**
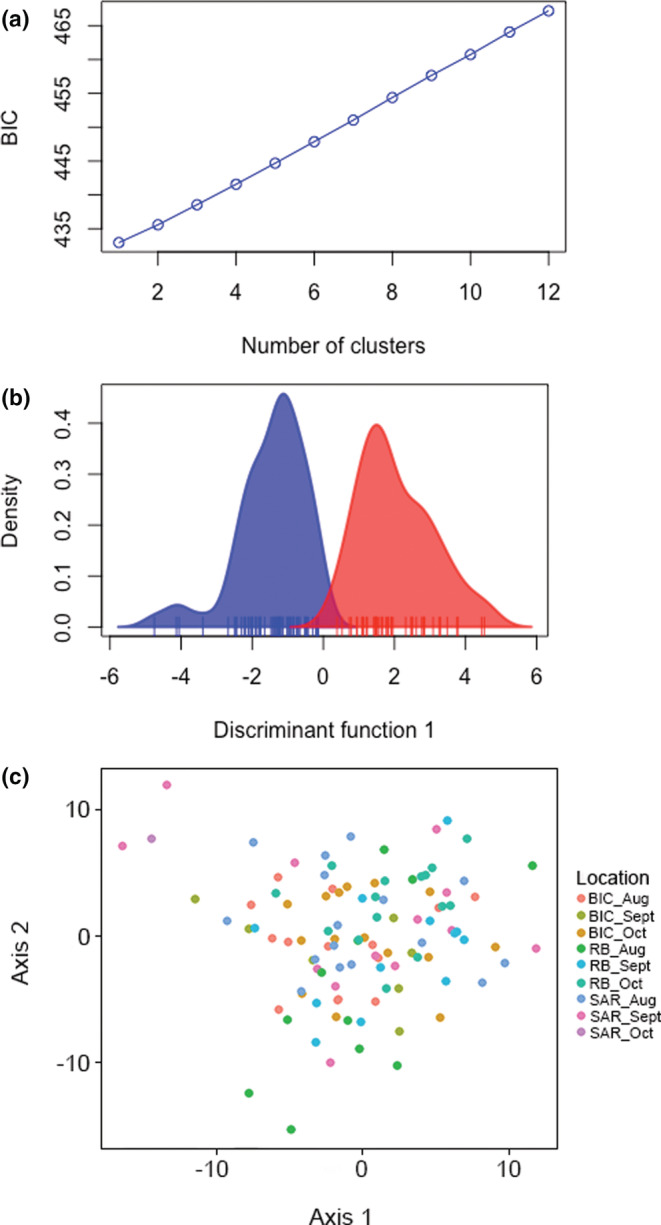
Genetic differentiation explored through (a) a scree plot showing the number of clusters and (b) first principal component plot resulting from the DAPC, as well as a (c) principal component analysis plot based on allele frequencies of all samples using pueruli settling into Bicheno (BIC), South Arm (SAR) and Recherche Bay (RB) during three consecutive winter months during 2012 with the neutral SNP panel.

**FIGURE 5:**
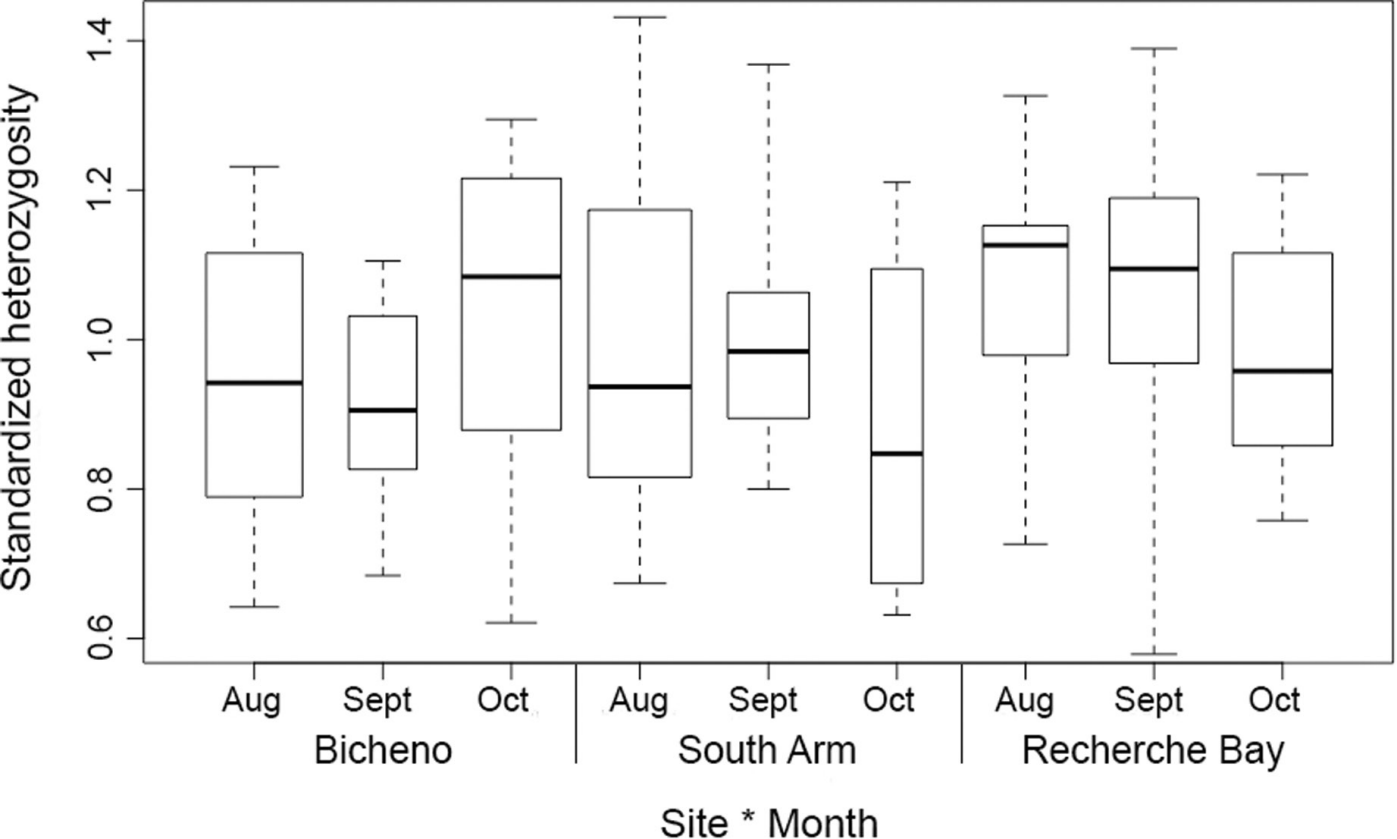
Median standardized individual heterozygosity (sh) at each site during each settlement month when analyzing the neutral SNP panel. Boxes represent quantiles, while whiskers represent minimum and maximum values.

There was no evidence of genetic differentiation in the neutral SNP panel across all sites and settlement months (global *F*_ST_ = −0.001, n.s., Figures 4 and 5). However, pueruli settling into Recherche Bay during August was genetically distinct from individuals settling into Bicheno and South Arm (Table 4), corresponding to the phenotypic differences between the sites described above. In addition, individuals settling into Recherche Bay during August were genetically distinct from those settling during October (Table 4).

**TABLE 4:** Pairwise *F*_ST_ values between each site and month of settlement for the neutral SNP panel

	BIC Aug	BIC Sept	BIC Oct	SAR Aug	SAR Sept	SAR Oct	RB Aug	RB Sept
BIC Sept	0.0027							
BIC Oct	0.0021	0.0089						
SAR Aug	0.0029	0.0010	−0.0008					
SAR Sept	0.0021	0.0069	0.0019	0.0005				
SAR Oct	0.0027	0.0061	−0.0089	−0.0059	−0.0029			
RB Aug	0.0043	**0.0166**	**0.0160**	**0.0142**	0.0021	0.0187		
RB Sept	−0.0055	0.0117	−0.0014	−0.0019	0.0021	0.0046	0.0028	
RB Oct	0.0009	0.0047	0.0026	0.0014	0.0055	−0.0081	**0.0205**	0.0015

*Note*: Significant differences after false discovery rate (FDR) correction are in bold.

Allele frequency variation was driven by the settlement site, although it accounted for a small proportion of the genetic variation between pueruli when analyzing the neutral SNP panel (Table 5).

**TABLE 5:** Analysis of molecular variance (AMOVA) results using genetic distance as a function of (1) settlement month and (2) settlement site using the neutral SNP panel.

Source of variation	df	SS	MS	Var	%	*p*	Pseudo‐*F*
(1) Settlement month							
Between Month	2	186.328	93.1638	−0.0042	−0.0041	.518	−4.14 E‐05
Between samples Within Month	101	9438.91	93.4546	−8.9028	−8.6981	1	
Within samples	104	11,571	111.26	111.2601	108.702	1	
Total	207	21196.3	102.398	102.3531	100		
(2) Settlement site							
Between Location	2	**210.319**	**105.16**	**.1731**	**.169**	**.033**	.0017
Between samples Within Location	101	9414.92	93.2171	−9.0215	−8.8091	1	
Within samples	104	11,571	111.26	111.2601	108.64	1	
Total	207	21196.3	102.398	102.4117	100		

*Note*: Significant differences are in bold.

On the other hand, the SNPs under putative positive selection exhibited a stronger overall level of genetic differentiation (Global *F*_ST_ = 0.0852, *p* = .0047). Pairwise *F*_ST_ values and AMOVA were not calculated for this SNP panel, since it was only comprised of two loci. Transcriptome sequences from the eyestalk, optical nerve, green gland, and hepatopancreas of *J. edwardsii* exhibited significant hits with one locus containing SNPs under putative positive selection. However, the transcriptome sequences could not be successfully annotated to any gene, possibly due to the short fragment size (i.e., 75 bp). Additionally, no significant hits between the panel of SNPs under putative positive selection and the *H. americanus* transcriptome were found.

**FIGURE 6:**
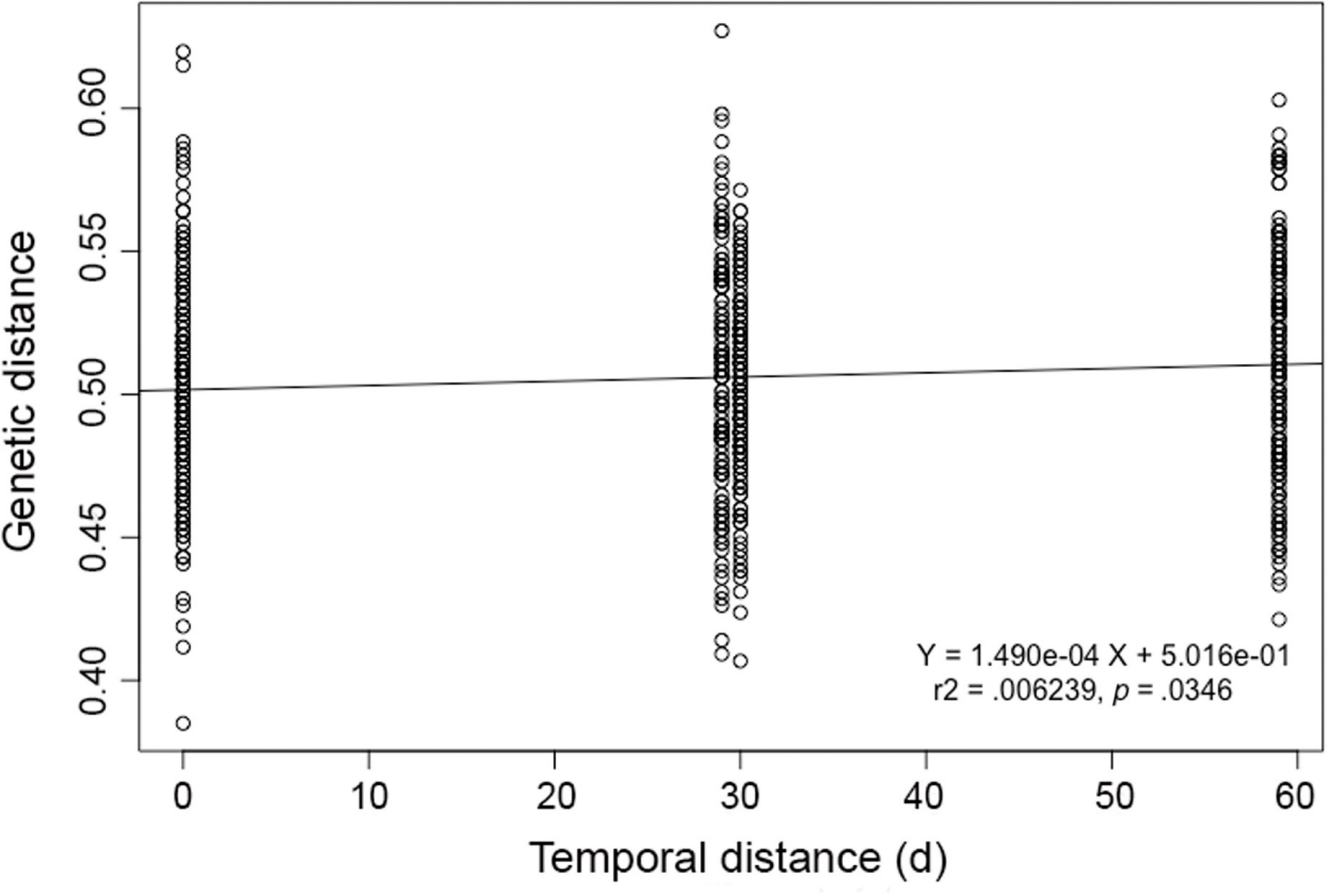
Relationship between genetic distance at the neutral SNP panel and temporal distance at Recherche Bay. On the x‐axis, zero corresponds to samples collected within the same month, 29 corresponds to genetic distance between individuals settling during August and September, 31 corresponds to genetic distance between individuals settling during September and October, 60 corresponds to genetic distance of individuals settling between August and October.

In support of the small F_ST_ differentiation between settlement months in Bicheno and South Arm, the isolation by time analyses showed no significant correlation between genetic distance and distance between recruitment time (Table 6). Similarly, the significant F_ST_ values found among the Recherche Bay sampling periods were confirmed by the significant Mantel test at this site; however, the *r* value was very low (*r* = 0.087), indicating the existence of a very low correlation. Pueruli settling into Recherche Bay during the same month was genetically more similar to each other than individuals settling one or two months apart, as shown by the slight but significant regression (Figure 6).

**TABLE 6:** Results of the Mantel tests between temporal and genetic distance at each settlement site using the neutral SNP panel.

Settlement site	*r*	*p*
Bicheno	−0.004	.5418
South Arm	−0.153	.9801
Recherche Bay	0.087	**.0346**

*Note*: Significant differences are in bold.

## 4 DISCUSSION

The present study examined pueruli size‐at‐settlement and genetic signatures during a winter settlement peak at three sites on the east coast of Tasmania, Australia. Significant differences in weight between settling pueruli were evident among the study sites. Pueruli settling into the northernmost site, Bicheno, were consistently smaller than those arriving at the other two sampling sites. Despite the overall low level of genetic differentiation at neutral markers, the southernmost site (Recherche Bay) was genetically divergent from the other sites and also exhibited temporal genetic divergence across settling cohorts.

### 4.1 Dispersal history driving phenotypic variation

The environmental history experienced by larvae influences their survival and development, and the resulting phenotypic variability is a general response contingent on the level of environmental stress experienced by larvae (González‐Ortegón & Giménez, 2014). For example, marine shrimp (*Palaemon serratus*) larvae exposed to different combinations of temperature, food, and salinity treatments exhibited a longer duration of development (increased number of instars) under suboptimal conditions (González‐Ortegón & Giménez, 2014). A similar trend was observed in experiments performed on larval European lobster, *H. gammarus*, and velvet crab, *Necora puber*, where limited food access delayed development by up to 25% depending on temperature (D'Urban Jackson et al., 2014). More specifically, variations in size‐at‐settlement, measured as puerulus carapace length, have been observed in *J. edwardsii* arriving during different years and seasons at the same site (Booth, 1979; Booth & Tarring, 1986). It has been observed that stage 1 pueruli settling during winter in the northwest of New Zealand were significantly bigger than those settling during other times of the year (Booth & Tarring, 1986). Also, in a three‐year study, Booth (1979) found significant differences in size‐at‐settlement of *J. edwardsii* arriving at one site in northeast New Zealand between most years and months of settlement. The authors attributed the phenotypic differences to: (1) different cohorts of phyllosoma metamorphosing into pueruli and settling simultaneously, and (2) to pueruli originating from different parental stocks (Booth, 1979). Here, we aimed to explore *J. edwardsii* pueruli differences in size‐at‐settlement further and investigate whether these could have a genetic origin. The lack of neutral genetic structure found in the present study indicates that it is unlikely that the phenotypic variation is directly linked to different larval sources. Thus, based on our results, we hypothesize that the observed phenotypic differences between pueruli settling into three sites in Tasmania during the study period were caused by the environmental history of settling individuals throughout dispersal.

The environment has profound effects on the metabolism and growth of larval lobsters, which can be carried over into subsequent stages (Green et al., 2014). *Jasus edwardsii* settling into east Tasmania can spend up to 2 years as phyllosoma larvae, encountering a large gradient of environmental factors (Bruce et al., 2007). Temperature and food availability are the main factors affecting the growth rates of larvae and can determine settlement success (Pechenik, 2006). Therefore, individuals that encountered temperature and food conditions that promoted growth during the late phyllosoma stage, when they are likely closer to settlement grounds, could have a higher accumulation of energy reserves and metamorphose into larger pueruli (Jeffs et al., 2001). If the significant differences in pueruli weight across sites were caused by differences in environmental conditions during development, the persistence of size differences across the study sites would require that larvae were traveling in cohorts and experiencing different temperature and nutrition regimes, for at least the last part of their larval life. Interestingly, collective dispersal has been previously proposed to occur in spiny lobsters (Funes‐Rodríguez et al., 2015; Iacchei et al., 2013; Villacorta‐Rath et al., 2018).

While larval cohesiveness could be a plausible mechanism of dispersal in *J. edwardsii*, individuals of different ages could also congregate near settlement grounds, spending only the last period of their PLD together. Late‐stage *J. edwardsii* phyllosoma has been observed to congregate beyond the shelf break in oceanic waters, possibly through a combination of oceanic advection and horizontal swimming (Chiswell & Booth, 1999). Differences in age‐at‐settlement and dispersal history could possibly result in differences in size‐at‐settlement in *J. edwardsii* found in the present study. A mixture of collective dispersal and convergence of larvae at later stages has been described in a reef fish cohort settling into the same site (Shima & Swearer, 2016). If this also occurs in *J. edwardsii*, then the genetic signature of settling individuals will be more randomly distributed, with no genetic divergence, as seen in Bicheno and South Arm but with a large variation in phenotypes of settlers due to divergent dispersal histories. Our data show that the total weight range (difference between maximum and minimum weight) varies across months within each sampling site, suggesting that settlers could have undergone different environmental histories. Oceanographic modeling would allow determining whether collective dispersal, larval cohesiveness close to settlement sites, or a combination of both could be driving the observed phenotypic, but no genetic differences were observed in the present study.

Finally, the temporal differences in pueruli weight could be attributed to differences in environmental conditions related to the time of hatching. A latitudinal gradient of the incubation period due to differences in temperature has been described in the Dungeness crab, *Cancer magister* (Shirley et al., 1987) where individuals hatching at lower temperatures were larger than those hatching in warmer waters. Also, if time and place of hatching can determine where larvae will be advected, this would result in different larval cohorts experiencing divergent environmental conditions. It is therefore possible that females spawning at different times of a reproductive season can produce offspring with slightly different phenotypes (Kunisch & Anger, 1984; Shirley et al., 1987). This could have been the case in the present study, where individuals recruiting during different months exhibited significant differences in weight‐at‐settlement. The same trend was observed in the green crab, *Carcinus maenas*, where larval size at metamorphosis and survival of the first crab instar under starvation varied significantly across four supply events (Rey et al., 2016). Individuals sampled earlier in the season exhibited smaller sizes than those sampled later, probably due to less favorable environmental conditions encountered by the former group (Rey et al., 2016).

The question that arises here is whether fitness in *J. edwardsii* settlers is related to size‐at‐settlement and can lead to successful recruitment. Although there is a general hypothesis that larger offspring are fitter than smaller offspring, the link between size and fitness is not completely understood (Giménez, 2006; Marshall et al., 2006). Larger individuals generally exhibit improved anti‐predatory behavior, are bolder, can forage for a longer time, and better resist periods of starvation (Dingeldein & White, 2016; Johnson et al., 2017). However, it is often the environment (biotic and abiotic factors) that ultimately provides the selective pressure and determines survival (Marshall et al., 2006). If smaller size represents a disadvantage for postsettlement survival, it would then result in fewer pueruli recruiting to the fishery and lower productivity from Bicheno compared with the other sampling areas studied here. This is actually the case. Average counts of puerulus at Bicheno are substantially higher than the other sites (Gardner, unpublished data), yet the Bicheno region has the lowest fishery productivity in Tasmania (Gardner et al., 2015). Therefore, it is possible that in *J. edwardsii*, smaller pueruli are less fit and less likely to recruit into the fishery.

### 4.2 Genetic differentiation at the southernmost site

Lack of neutral genetic differentiation between sampling events suggests that populations sourcing pueruli into the east coast of Tasmania during winter months are genetically homogenous; however, there is a slight indication of genetic differentiation at the southernmost site. Lack of overall genetic divergence but significant differences at smaller spatial or temporal scale is typical of chaotic genetic patchiness (Eldon et al., 2016), which is inline with previous evidence of chaotic genetic patchiness in spiny lobsters (reviewed by Silva et al., 2019).

The significant genetic differentiation between pueruli settling during August and October at Recherche Bay could indicate the existence of different populations (or groups of individuals) sourcing pueruli during the winter months. The slight differences in individual weights between months found and size‐at‐settlement could be a signature of each of these populations. However, the small sample size of the present study precludes the possibility of proving that the observed genetic divergence at Recherche Bay is due to chaotic genetic patchiness or IBT.

## 5 CONCLUSIONS

The observed variation in size‐at‐settlement of pueruli observed in the present study is likely due to phenotypic variability during dispersal, rather than the genetic signature of the parental stock. The hypothesis of cohorts of larval *J. edwardsii* maintaining cohesiveness and experiencing the same environmental conditions is a likely explanation for the observed phenotypic differences between settlement months and sites in the lack of overall neutral population differentiation. The lack of neutral population differentiation suggests that neither the time nor place of reproduction acts as a biological barrier to the dispersal of *J. edwardsii* populations on the east coast of Tasmania. A larger panel of markers under putative positive selection and a larger sample size could help determine whether observed phenotypic differences across sites and months were driven by selection in response to environmental factors.

## AUTHOR CONTRIBUTIONS

**Cecilia Villacorta‐Rath:** Conceptualization (equal); data curation (lead); formal analysis (lead); investigation (lead); methodology (lead); writing – original draft (lead). **Bridget S Green:** Conceptualization (lead); funding acquisition (lead); methodology (lead); project administration (lead); resources (lead); writing – review and editing (equal). **Caleb Gardner:** Conceptualization (supporting); funding acquisition (lead); investigation (supporting); methodology (supporting); project administration (lead). **Nicholas P Murphy:** Formal analysis (supporting); investigation (supporting); resources (equal); writing – review and editing (equal). **Carla A. Souza:** Data curation (equal); formal analysis (supporting); investigation (supporting). **Jan Strugnell:** Conceptualization (equal); formal analysis (equal); investigation (equal); methodology (equal); project administration (lead); resources (lead); writing – review and editing (equal).

## CONFLICT OF INTEREST

The authors declare that they have no competing interests.

## Data Availability

Genotype (VCF) file and reference loci sequences are available through Dryad, doi:10.5061/dryad.5mkkwh792.
